# Thrombotic Thrombocytopenic Purpura Associated with Pazopanib

**DOI:** 10.1155/2018/4327904

**Published:** 2018-07-02

**Authors:** Umer Syed, Kramer J. Wahlberg, Daniel R. Douce, Julian R. Sprague

**Affiliations:** ^1^Department of Internal Medicine, University of Vermont Medical Center, Burlington, VT, USA; ^2^Department of Hematology and Medical Oncology, University of Vermont Medical Center, Burlington, VT, USA

## Abstract

A 76-year-old male with metastatic renal carcinoma on day 24 of pazopanib was admitted with complaints of emesis, confusion, and hematuria. Laboratory testing showed acute kidney injury, hyperbilirubinemia, and thrombocytopenia. Scattered schistocytes were seen on peripheral smear, and he was diagnosed with thrombotic microangiopathy (TMA). He was started on daily, one-volume plasma exchange with rapid improvement in thrombocytopenia. ADAMTS13 activity returned as undetectably low with no inhibitor detected. After cessation of plasmapheresis, repeat ADAMTS13 activity returned as normal. Unfortunately, his platelet count started to downtrend within four days after developing septicemia thought to be due to a catheter-associated infection. He was placed on comfort care measures after discussion with his family. An autopsy listed the major cause of death as metastatic renal cell carcinoma. According to two separate systematic reviews, there have been no cases of proven drug-induced TMA where decreased ADAMTS13 activity was the identified mechanism. While pazopanib is also associated with TMA, this unique case suggests a novel potential mechanism for TMA associated with pazopanib and brings forth “drug-induced thrombotic thrombocytopenic purpura” that quickly responds to plasmapheresis as a possible new diagnostic entity requiring prompt recognition and treatment.

## 1. Introduction

Thrombotic thrombocytopenic purpura (TTP) is a life-threatening thrombotic microangiopathy (TMA) characterized by platelet microthrombosis of the microvasculature due to decreased activity of the ADAMTS13 enzyme responsible for cleaving von Willebrand factor. Impaired ADAMTS13 activity can be due to hereditary enzyme deficiency or due to the acquisition of an autoantibody inhibitor [1].

This has been differentiated from drug-induced TMA (DITMA) which is defined as microangiopathic hemolytic anemia, thrombocytopenia, and microvascular thrombosis with characteristic vasculature abnormalities [2, 3]. DITMA has generally been defined to have two different mechanisms: immune-mediated and toxicity-mediated. In immune-mediated DITMA, antibodies are formed that bind with numerous cells including platelets, neutrophils, and endothelial cells and causes the adverse effect which is independent of dose and occurs within 2-3 weeks of drug exposure [4]. One example of this is TMA associated with quinine. In reactions where typical features of immune-mediated TMA are not present or the reaction occurs gradually (weeks to months), the drug is usually assigned to the toxicity-mediated TMA category [2]. Chemotherapeutics may lead to TMA via this mechanism. We present a case of a patient who presented with inhibitor-negative TTP following the initiation of a chemotherapeutic agent for renal cell carcinoma (RCC).

## 2. Case Presentation

A 76-year-old male with past medical history of grade 3 RCC on day 24 of pazopanib after a left radical nephrectomy, atrial fibrillation, coronary artery disease, hyperlipidemia, obesity, and obstructive sleep apnea presented with fatigue, dyspnea, hematuria, and confusion.

He initially was diagnosed with RCC four months prior to admission (PTA) in the setting of hematuria. Two days PTA, he presented to an outside hospital emergency department with complaints of nausea and emesis and was admitted overnight for intravenous hydration and discharged with mild improvement in symptoms. He subsequently presented to his medical oncology clinic for an acute visit on the day of admission with progressive symptoms and new confusion and was admitted to the inpatient hematology service.

Upon admission, the patient was found to be hemodynamically stable and febrile with temperature of 37.8°C on admission and 38.3°C on hospital day 3. Exam was significant for drowsiness, irregularly irregular heart rhythm, and bilateral lower extremity venous stasis changes.

Labs were significant for acute thrombocytopenia (32 × 10^9^/L, anemia (hemoglobin 12.6 gm/dL)), LDH 2001 U/L, fibrinogen 652 mg/dL, normal INR/PTT, elevated transaminases (AST 113 U/L and ALT 147 U/L), acute kidney injury (creatinine 1.59 mg/dL from baseline of 1.19 mg/dL), hyperbilirubinemia (2.2 mg/dL), and elevated LDH (2001). Haptoglobin was noted to be normal (135) on admission but was noted to downtrend to lower limit of normal (41) on day two of admission.

Scattered schistocytes were seen on peripheral smear, and he was diagnosed with thrombotic microangiopathy (TMA). Pazopanib was held, and he was subsequently started on daily, one-volume plasma exchange with rapid improvement in thrombocytopenia.

**Figure 1:**
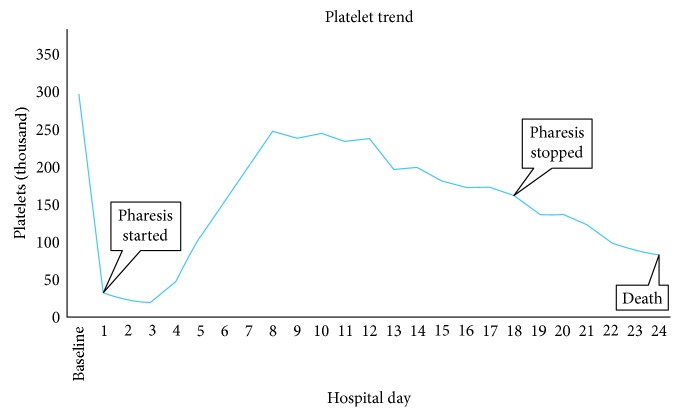
Graphic trend of platelet count over the course of hospitalization. Baseline platelets were normal (298 × 10^9^/L) 3 weeks PTA and found to be acutely low at 32 × 10^9^/L on admission (hospital day 1) with recovery to 248 × 10^9^/L on hospital day 8 following plasmapheresis.

The platelet count normalized (Figure 1), and ADAMTS13 activity returned as undetectably low with no inhibitor detected by mixing studies (ADAMTS13 assay FRETS-VWF73 via the Blood Center of Wisconsin) [5]. Plasmapheresis was continued for 19 days, as he had become febrile and confused on days when plasmapheresis was held. Repeat ADAMTS13 activity level two days after plasmapheresis was stopped and returned as normal. Platelet count initially remained stable after stopping plasmapheresis, but then started to downtrend within four days. He developed arrhythmias and catheter-related septicemia. Following discussion with family, care was transitioned to comfort-directed treatment plan. An autopsy listed the major cause of death as metastatic renal cell carcinoma.

## 3. Discussion

Pazopanib (Votrient®) is a tyrosine kinase inhibitor, which limits tumor growth by inhibiting growth factors resulting in inhibition of angiogenesis. In the pazopanib FDA package insert from Novartis, the side-effect profile describes “TMA” occurring in 7/977 patients during phase 3 trials [6]. Per follow-up discussion with Novartis, no additional clinical data are known about these patients with TMA, specifically whether they were diagnosed with TTP or hemolytic-uremic syndrome (HUS). Of these patients, six had TMA diagnosed within ninety days of treatment initiation and all had resolution with discontinuation of the drug. Nonetheless, no mechanism of this pazopanib-associated TMA has been defined, and a literature review did not reveal any reports of similar occurrences. The most common side effects were noted to be hypertension, fatigue, weight loss, diarrhea, leukopenia, and elevated transaminases. The half-life of pazopanib is approximately 31 hours [7, 8].

On literature review, there have been a plethora of case reports describing potential DITMA, drug-induced TTP (DI-TTP), and drug-induced HUS. However, according to two separate systematic reviews evaluating case reports involving 684 patients, there have been no cases of proven drug-induced TMA where decreased ADAMTS13 activity was the identified mechanism [2, 3]. Thus, DI-TTP has been disregarded as unique diagnostic entity.

While pazopanib is also associated with TMA, this unique case suggests a potential novel mechanism for pazopanib-associated TMA in the setting of undetectable ADAMTS13 activity and brings forth “drug-induced TTP that quickly responds to plasmapheresis” as a new diagnostic entity requiring prompt recognition and treatment.

This is supported by the fact that the patient's ADAMTS13 activity level was undetectable prior to treatment (plasma exchange and drug cessation) in the absence of a detectable inhibitor and subsequently improved to normal after treatment. While no ADAMTS13 activity levels were known prior to this episode of TTP, it would be exceedingly unlikely for this 76-year-old patient to have hereditary TTP without experiencing an acute episode earlier in life. Finally, there have been isolated case reports describing DI-TTP following initiation of drugs including interferon and thienopyridine antiplatelet agents (ticlopidine and prasugrel), among others [9, 10]. In essentially all of these reported cases, patients were found to develop severely reduced ADAMTS13 activity in the setting of a detectable inhibitor.

Further studies are needed to evaluate the exact mechanism by which pazopanib might lead to TTP. In the age of immunotherapies where many of the side effects are discovered initially during patient treatment, this case shows another such side effect requiring prompt recognition and treatment.
